# Supplementary material for: The DNA binding landscape of the maize AUXIN RESPONSE FACTOR family
Source: Nat Commun. 2018 Oct 30;9:4526. doi: 10.1038/s41467-018-06977-6 (PMC6207667; doi:10.1038/s41467-018-06977-6)
Supplement: Supplementary file 1 — Supplementary Information [file 41467_2018_6977_MOESM1_ESM.pdf]

THE DNA BINDING LANDSCAPE OF THE MAIZE AUXIN RESPONSE FACTOR FAMILY

Galli et al.

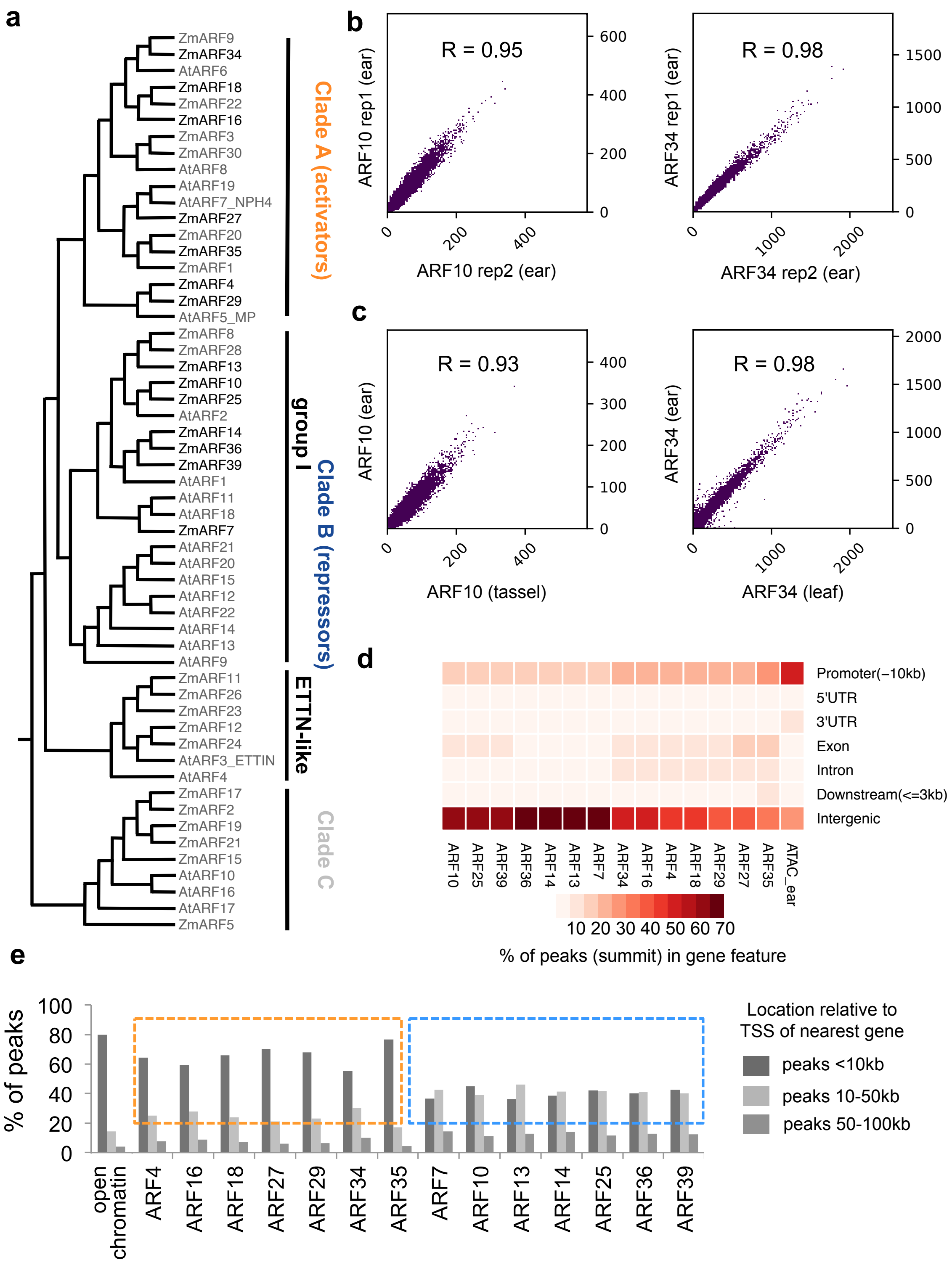

Supplementary Figure 1. ARF DAP-seq peaks are reproducible and located throughout the genome. a. Neighbor joining cladogram based on percent amino acid identity of the 33 expressed maize ARFs (ZmARF) and 22 Arabidopsis ARFs (AtARF). ZmARFs presented in this study are in bold. **b.** Scatterplots showing correlation between replicate DAP-seq experiments for three different ARFs **c.** Scatterplots showing correlation between replicate DAP-seq experiments performed with genomic DNA from different tissues. **d.** Percentage of peaks that are located within 10kb, 10-50kb, and 50-100kb of a gene body. Clade A ARFs are boxed in orange, clade B ARFs are boxed in blue. Open chromatin dataset corresponds to the ear ATAC-seq dataset. **e.** Heatmap showing percentage of peaks from each ARF dataset that are found in different genomic features. Promoter is defined as 10kb upstream of the TSS. Downstream is defined as 3kb downstream of the transcription termination site.

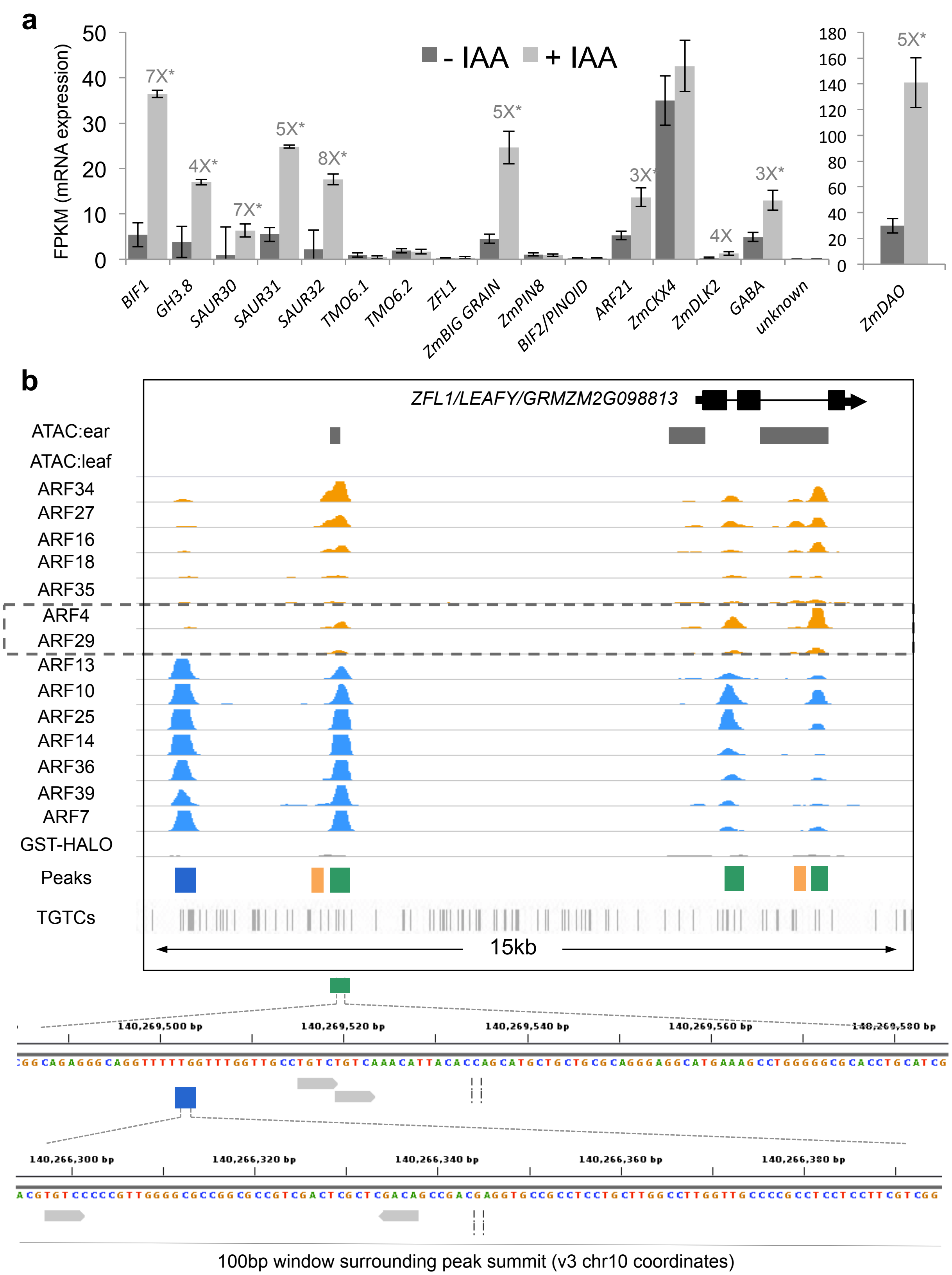

Supplementary Figure 2. ARF target genes include auxin regulated genes. a. mRNA expression values (FPKM) of mock treated (dark gray) or auxin induced (light gray) samples for selected genes shown in genome browser screenshots. Fold change values are indicated. Asterisk represents a FDR < 0.05. Error bars = s.d. **b.** Genome browser screenshots of ARF peaks at the *ZEAFLORICAULA1* (*ZFL1*) locus, the maize co-ortholog of Arabidopsis *LEAFY*. Bottom panels show sequence view of 100bp region surrounding peak summit. TGTC motifs present in the 100bp region are shown as gray bars. Clade A peaks, orange; clade B peaks, blue. Peaks track shows peaks called by GEM. Gray vertical lines in TGTCs track mark TGTC instances. Regions of accessible chromatin in immature ears and young leaves as determined by ATAC-seq are depicted as gray bars.

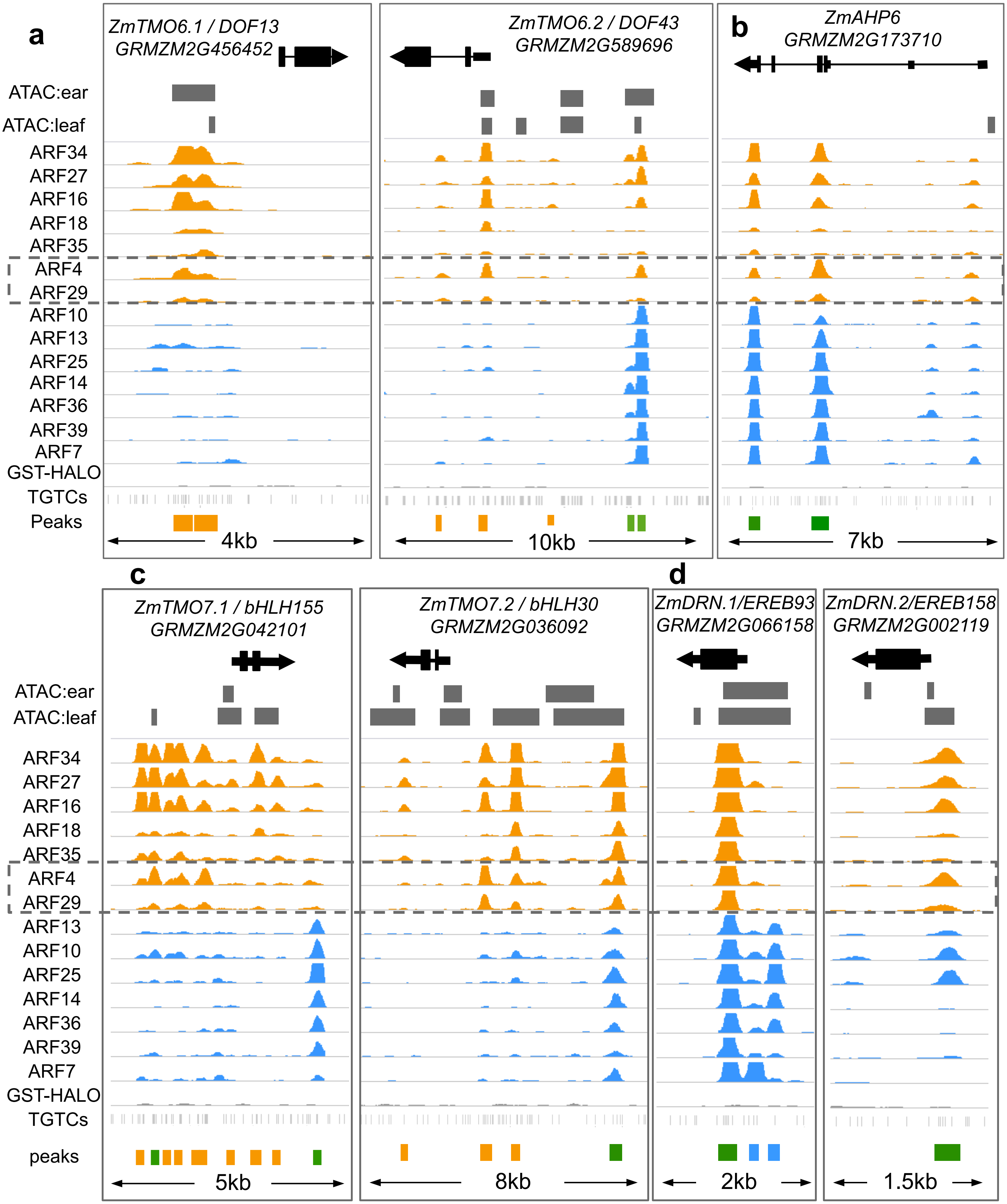

Supplementary Figure 3. Conserved ARF target genes. a. ARFs bind maize homologs of *TARGET OF MONOPTEROS6* (*TMO6*). ARF4 and ARF29 (dashed gray box) are the maize co-orthologs of MP. **b.** Maize homolog of AtAHP6 **c.** Two maize homologs of *TARGET OF MONOPTEROS7* (*TMO7*) **d.** Two maize homologs of *DORNROSCHEN* (*DRN*). Clade A peaks, orange; clade B peaks, blue. Peaks track shows peaks called by GEM. Gray vertical lines in TGTCs track mark TGTC instances. Regions of accessible chromatin in immature ears and young leaves as determined by ATAC-seq are depicted as gray bars.

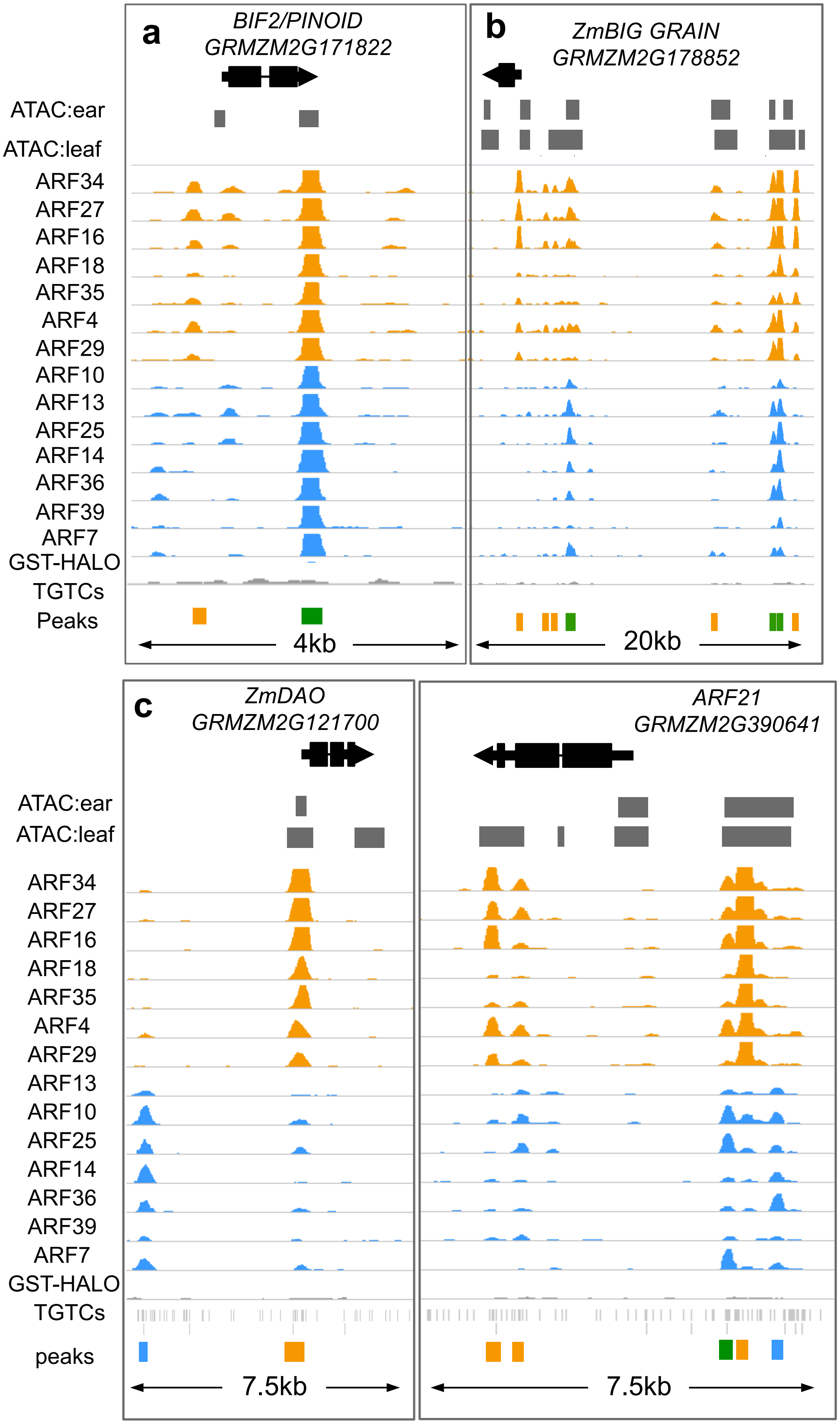

Supplementary Figure 4. ARF target genes include auxin related genes. a. The *BIF2/PINOID* locus involved in polar auxin transport. **b.** An auxin transport-related gene locus, the maize homolog of the rice BIG GRAIN1. **c.** Two auxin related genes not known to be directly bound by ARFs. Clade A peaks, orange; clade B peaks, blue. Peaks track shows peaks called by GEM. Gray vertical lines in TGTCs track mark TGTC instances. Regions of accessible chromatin in immature ears and young leaves as determined by ATAC-seq are depicted as gray bars.

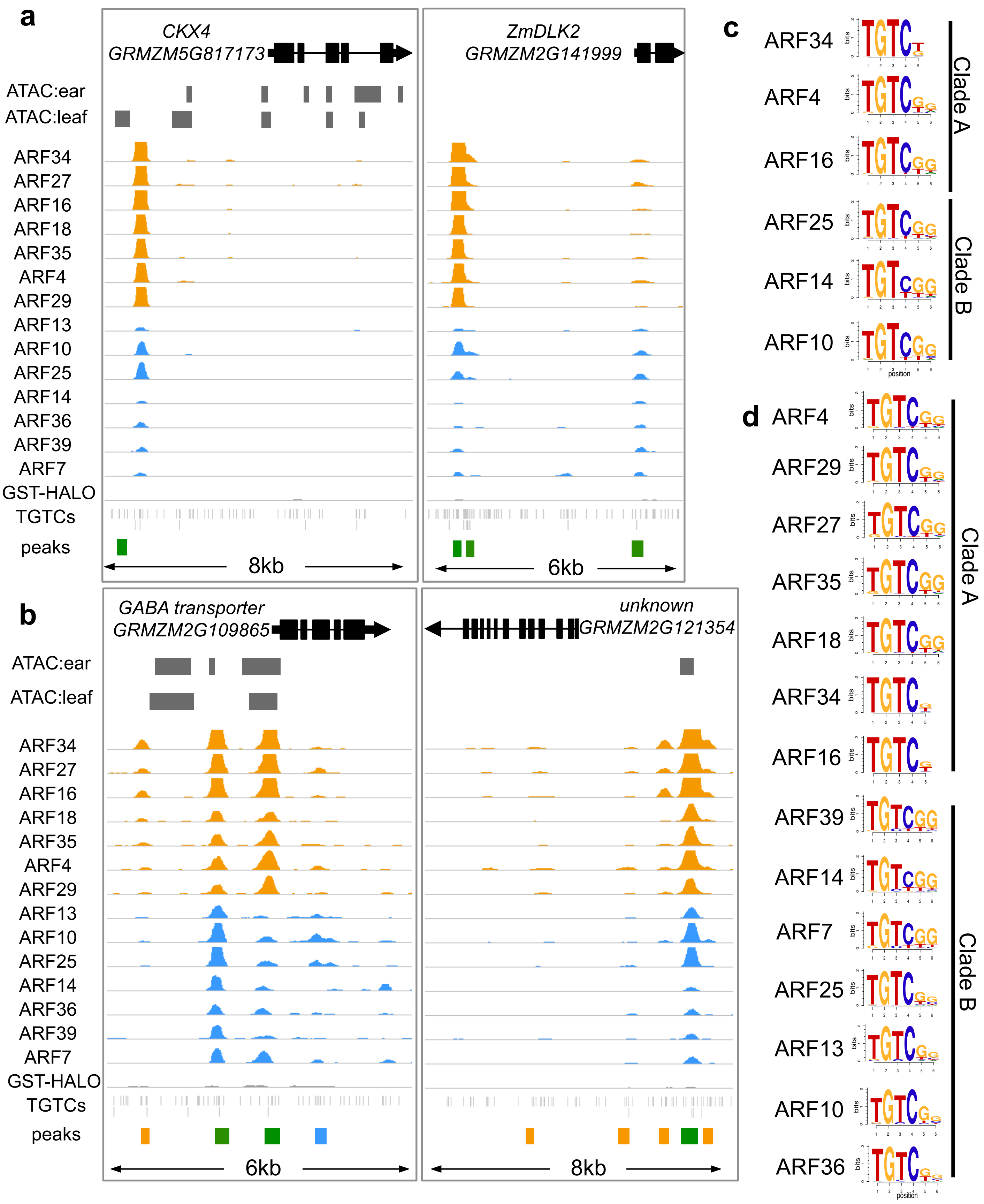

Supplementary Figure 5. **a.** Two hormone related genes bound by ARFs. **b.** Two uncharacterized genes bound by ARFs. Clade A peaks, orange; clade B peaks, blue. Peaks track shows peaks called by GEM. Gray vertical lines in TGTC instances track mark TGTC instances. Regions of accessible chromatin in immature ears and young leaves as determined by ATAC-seq are depicted as gray bars. **c.** Most enriched motif from Arabidopsis genomic DNA bound by maize ARFs. **d.** Most enriched motif in maize genomic DNA when only reads mapping to unique locations are used.

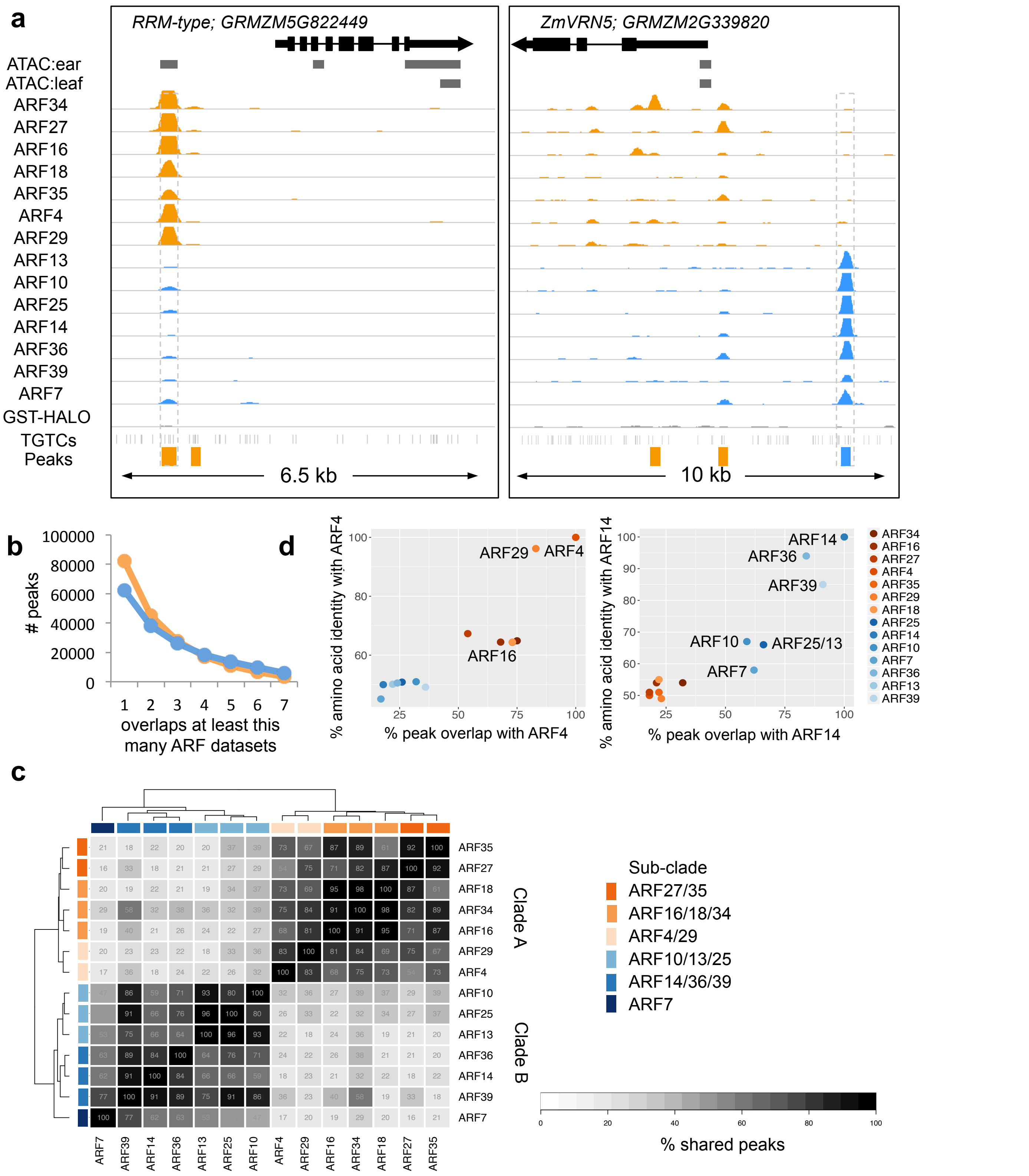

Supplementary Figure 6. Shared and unique features of ARF binding. a. Genome browser screenshot showing example of clade A-only peaks (left panel, orange) and clade B-only peaks (right panel, blue) at the *GRMZM5G822449* and *ZmVRN5* (*GRMZM2G339820*) loci. **b**. Number of unique peaks found in each of the seven clade A (orange) and clade B (blue) datasets as determined by DiffBind. **c.** Heatmap showing percentage of direct peak overlap between each ARF dataset. **d**. Scatterplots showing relationship between percent amino acid identity and percent peak overlap with ARF4 (clade A; left) and ARF14 (clade B; right).

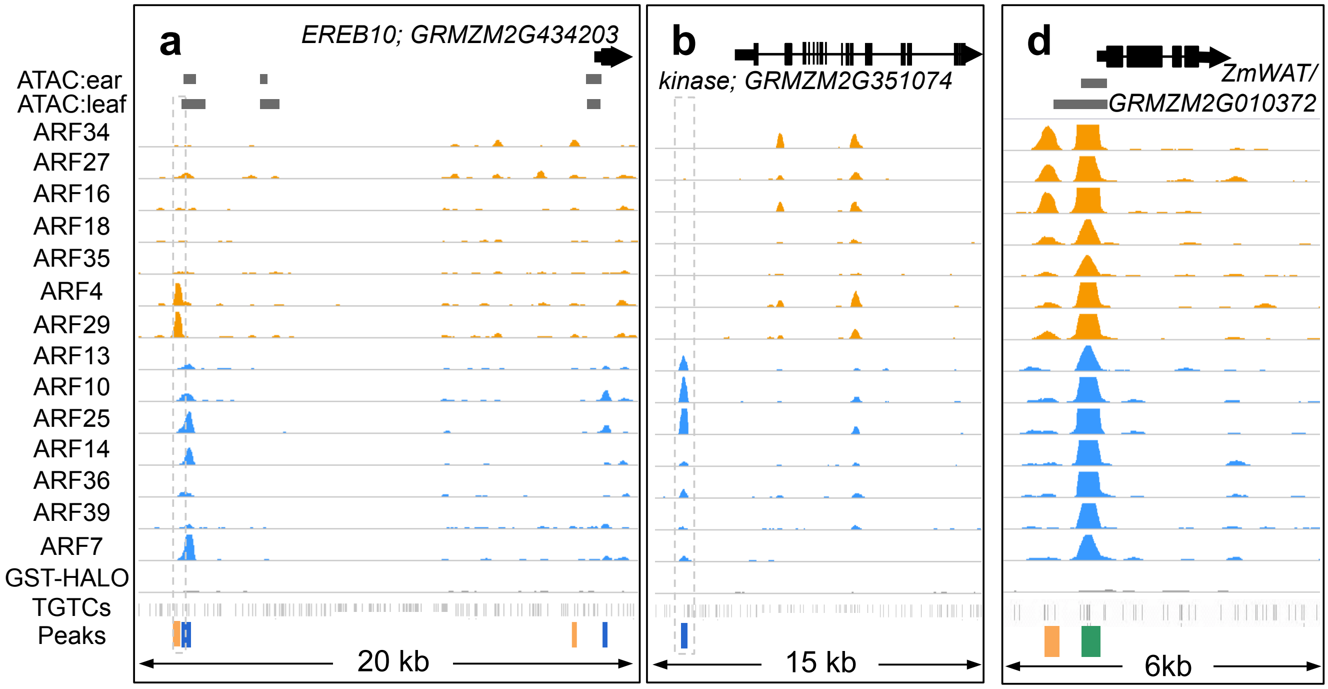

c sub-clade	class	# sub-clade specific peaks	% total peaks (124,530)	top motif	E score
ARF4, ARF29 (AtMP)	A	519	0.4%	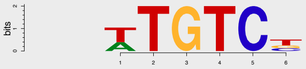	1.5e-070
ARF16, ARF18, ARF34 (AtARF6)	A	9516	7.6%	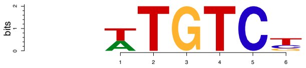	1.2e-1118
ARF27, ARF35 (AtNPH4)	A	119	0.1%	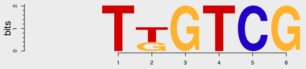	9.7e-027
ARF14, ARF36, ARF39 (AtARF1)	B	1047	0.8%	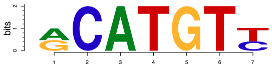	2.9e-045
ARF10, ARF13, ARF25 (AtARF2)	B	6717	5.4%	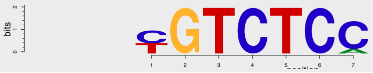	2.6e-1371
ARF7 (AtARF18)	B	8120	6.5%	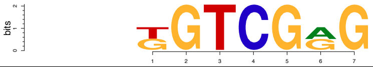	9.7e-264

e

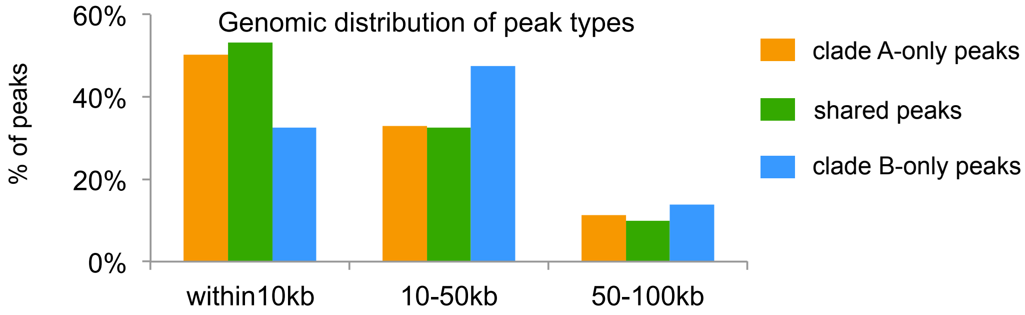

Supplementary Figure 7. Sub-clade specificity. a. Genome browser screenshot showing an ARF4 and ARF29 sub-clade specific peak (orange) at the *EREB10* locus. **b.** An ARF10, ARF13, ARF25 clade-specific peak (blue) at the *GRMZM2G351074* locus encoding an uncharacterized kinase. **c.** Table showing number of sub-clade specific peaks and results of motif enrichment analysis. The closest Arabidopsis homolog is indicated for each maize sub-clade. **d.** Genome browser screenshot showing shared peak at the maize *WALLS ARE THIN* (*ZmWAT*) locus. **e.** Percentage of clade A-only, clade B-only and shared peaks that are located within 10kb, 10-50kb, and 50-100kb of a gene body.

a

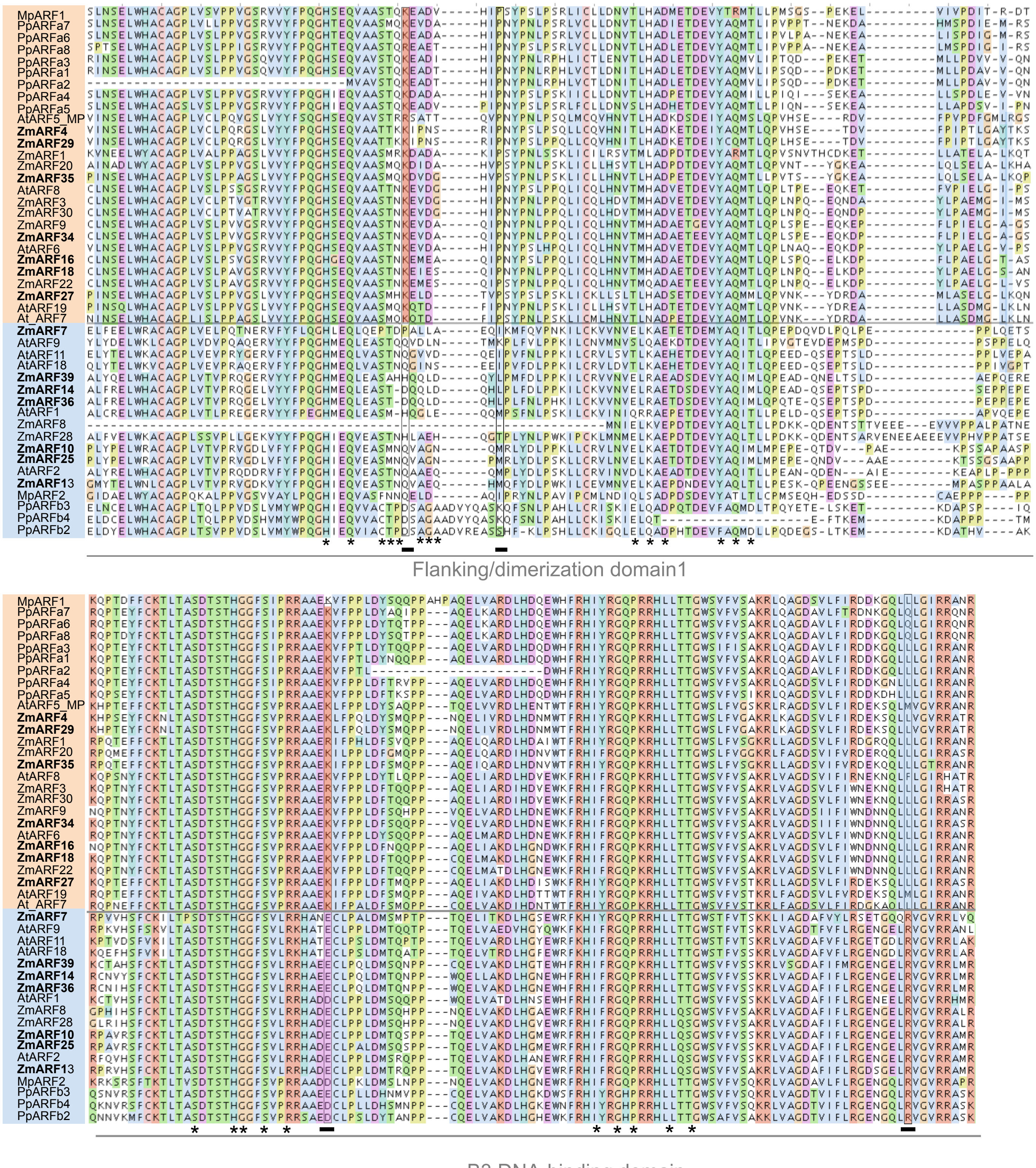

Supplementary Figure 8. Conservation of ARF DNA binding domain. a,b. Amino acid alignment of the extended DNA binding domains of maize (Zm), Arabidopsis (At), Physcomitrella (Pp), and Marchantia (Mp) ARFs. Flanking domain and dimerization regions correspond to those described in Boer et al., 2014 [1] and Guilfoyle 2015 [2]. Asterisks in the B3 DBD region indicate DNA contacting residues described for AtARF1 by Boer et al., 2014 [1]. Asterisks in the flanking and dimerization domain indicate residues at the dimer interface. Amino acids conserved in all four species for either clade A or clade B ARFs are indicated by a small black bar.

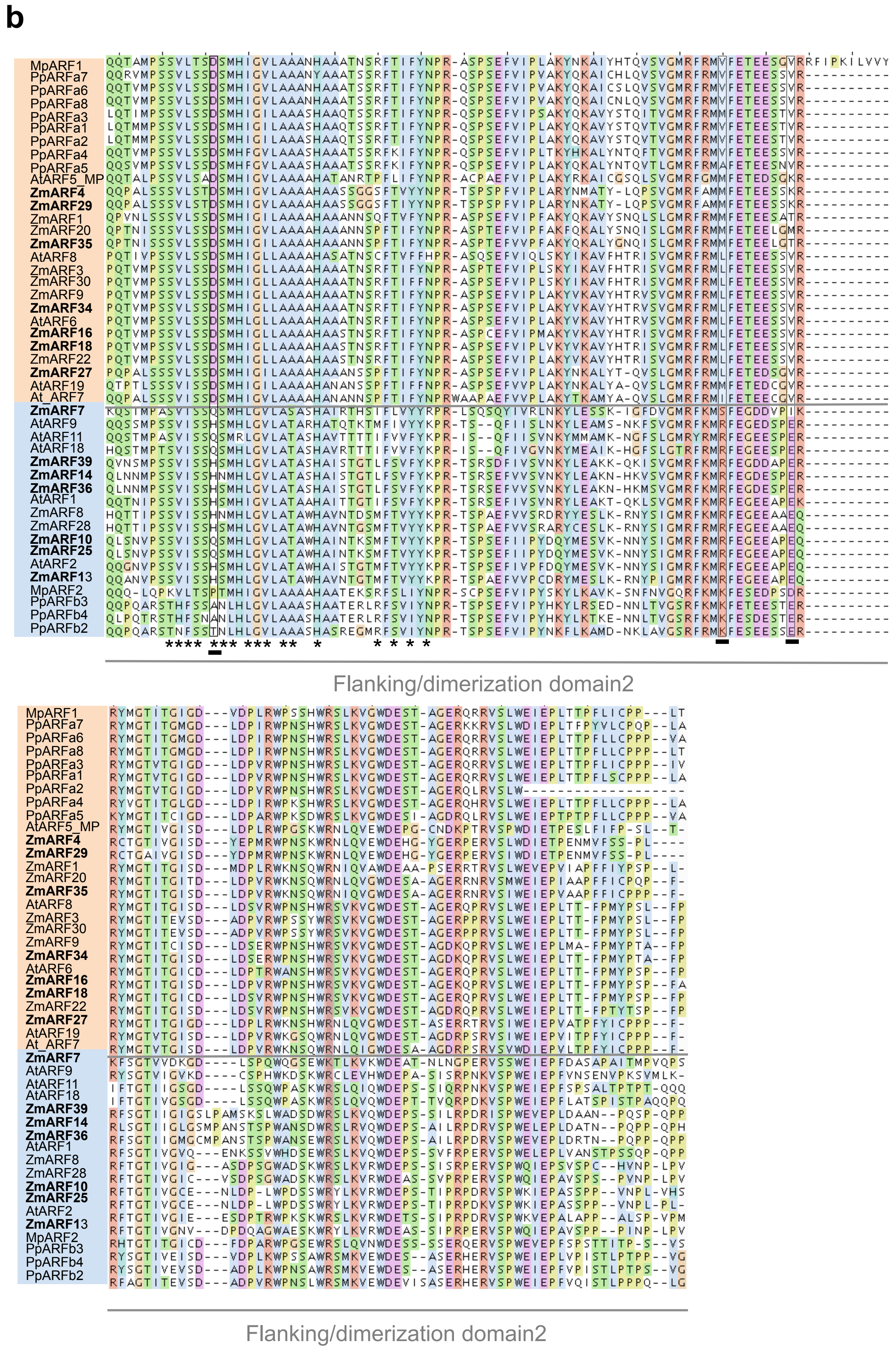

Supplementary Figure 8 continued. Conservation of ARF DNA binding domain.

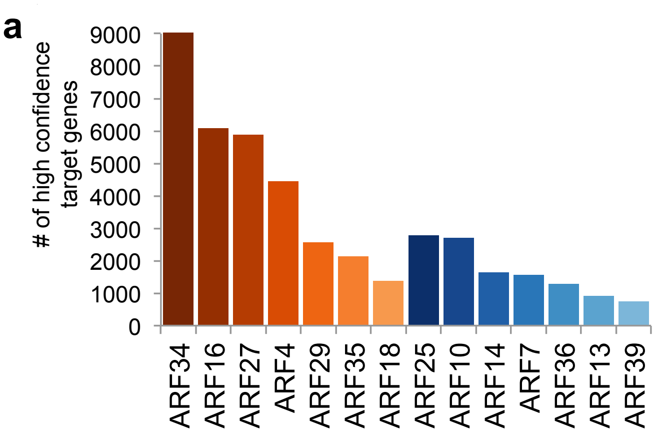

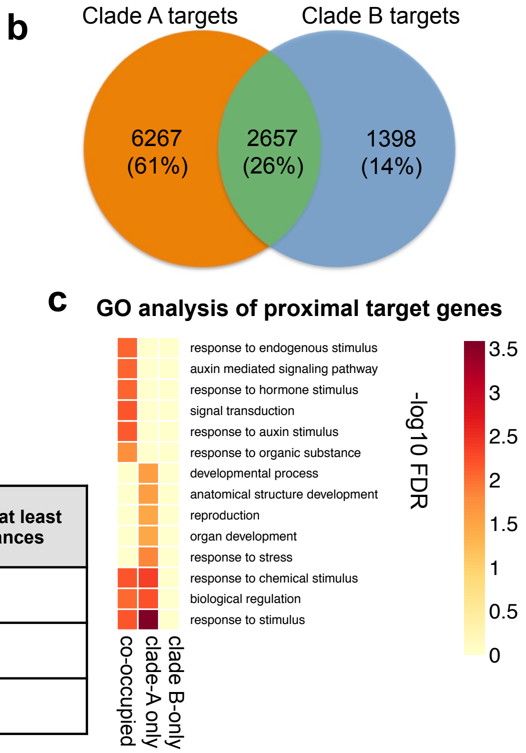

d

motif	number of motif instances in genome	% of instances bound by ARFs	% of peaks with at least two motif instances
TGTC	15,297,200	0.8%	55-86%
TGTCGG	764,640	2.6%	3-10%
TGTCTC	981,218	2.2%	1-3%

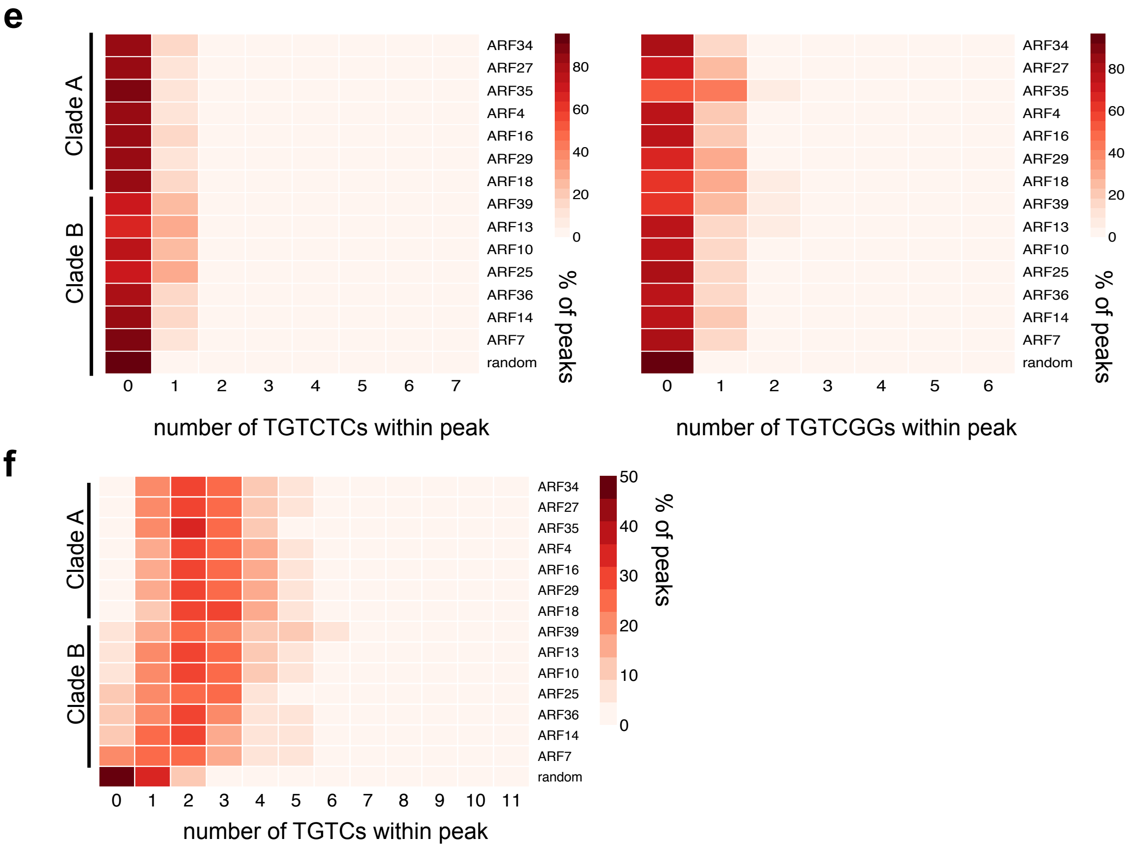

Supplementary Figure 9. ARF target genes contain TGTC repeats. a. Number of high confidence proximal target genes for each ARF. **b**. Venn diagram showing overlap among clade A and clade B high confidence proximal target genes. **c.** GO analysis of high confidence proximal target genes of clade-A only, clade B-only, and co-occupied loci. **d.** Table showing the frequency of TGTC containing sequence instances in the genome and the total percentage that are bound by ARFs. **e.** Heatmap showing the percentage of ARF peaks containing the indicated number of TGTCTC or TGTCGG motifs. **f.** Heatmap showing the percentage of ARF peaks containing TGTC.

a

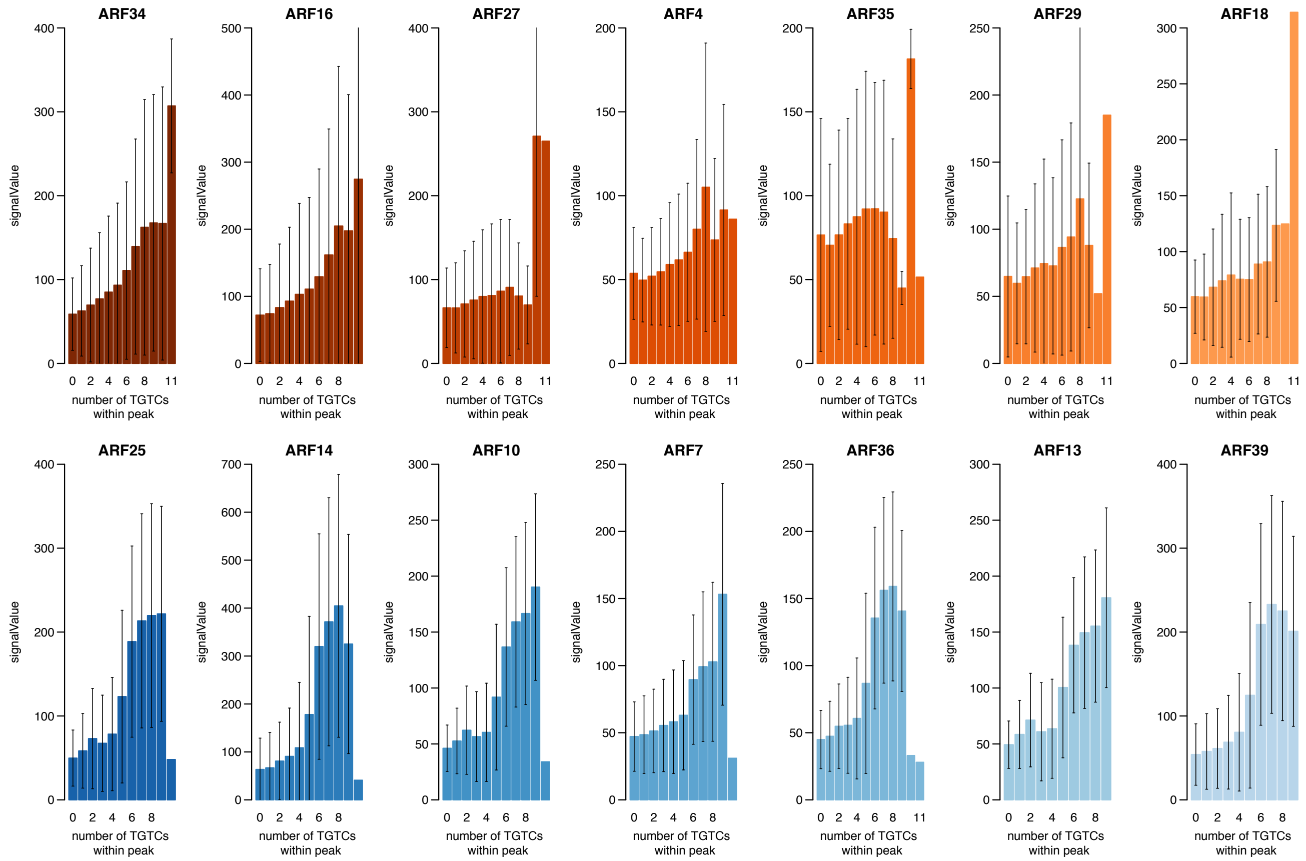

b

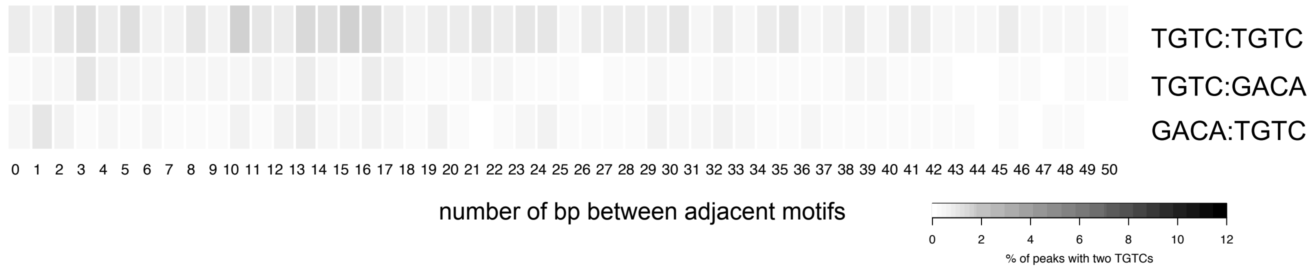

c

Dyad motif	Spacing	ARFs	Max E-score
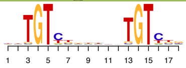	6	ARF18	3.0e-26
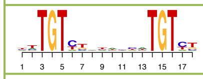	7	ARF16,18,34	1.7e-24
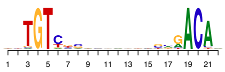	11	ARF10,13,14, 25,36,39	4.5e-22
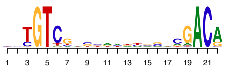	12	ARF10,13,14, 25,36,39,7 ARF27,29,35	1.2e-26

Dyad motif	Spacing	ARFs	Max E-score
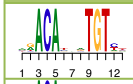	3	ARF34	5.6e-215
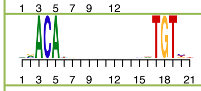	11	ARF14,36	6.2e-23
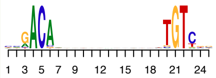	13	ARF18	6.7e-23
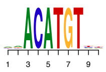	0	ARF4 ARF10,14, 36,39	8.0e-25

Supplementary Figure 10. ARF peak strength correlates to the number of TGTC elements. a. Average peak signal intensity for peaks containing different numbers of TGTCs. Error bars show standard deviation. **b.** Percentage of 4000 randomly selected genomic regions containing two adjacent TGTCs in the TGTC:TGTC, TGTC:GACA, or GACA:TGTC orientation that contain the indicated number of nucleotides separating adjacent motifs. **c**. Spaced pairs of TGTG repeats identified in ARF datasets using *de novo* dyad motif discovery analysis.

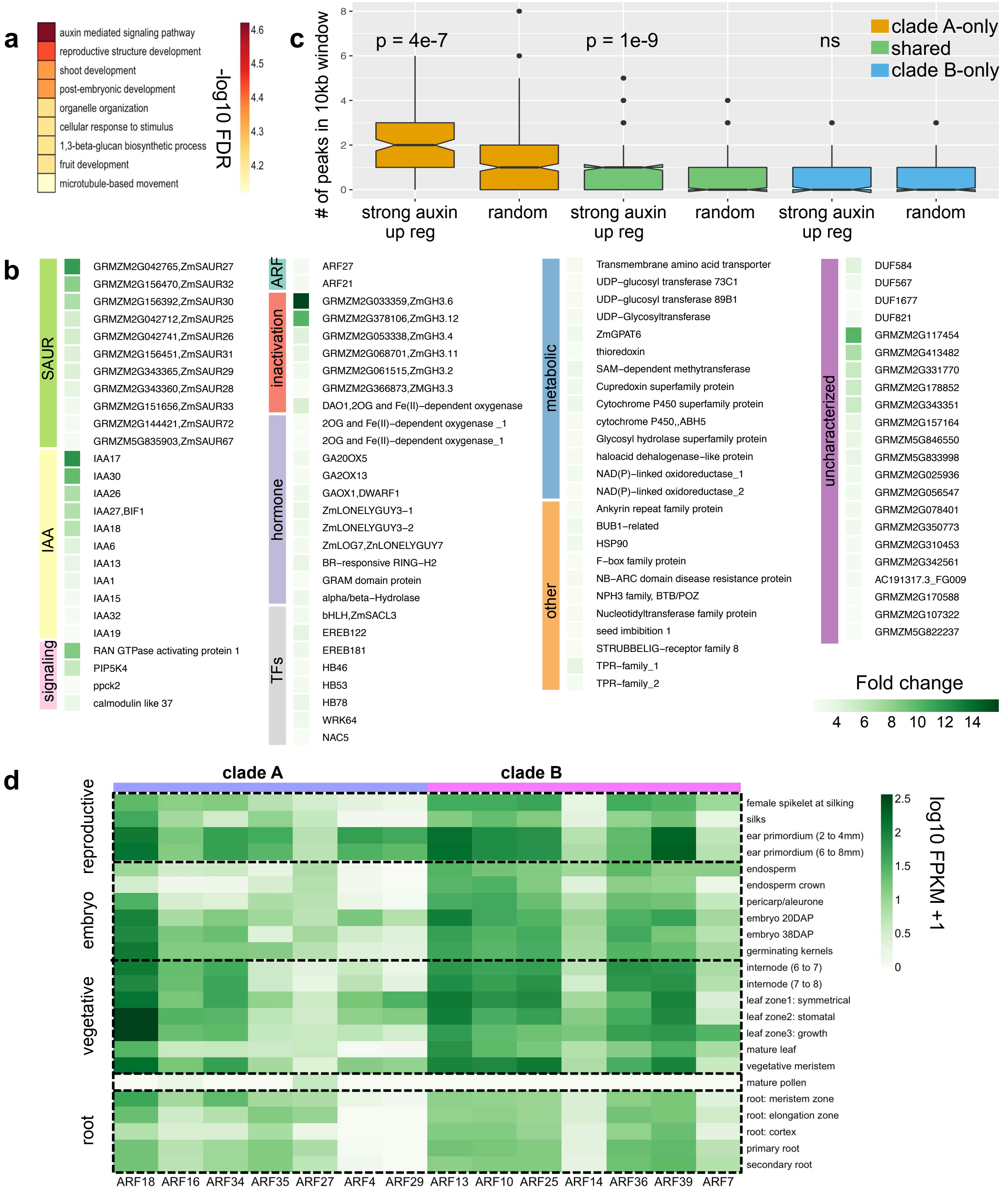

Supplementary Figure 11. Auxin induced gene analysis. a. GO enrichment analysis of auxin induced genes. **b**. Gene names and functional categories of the top 100 auxin induced genes. **c**. Distribution of the number of peaks located within 10kb of auxin induced or randomly selected (n=350) genes according to clade A-only, clade B-only, or shared peak categories. p-values determined by two-sided t-test; ns, not significant; central line shows median, upper and lower hinges show first and third quartiles, notches show 95% confidence intervals. **d.** *ARF* genes show a broad range of expression across various tissues. Normalized FPKM RNA-seq data from Walley et al. 2016 [3].

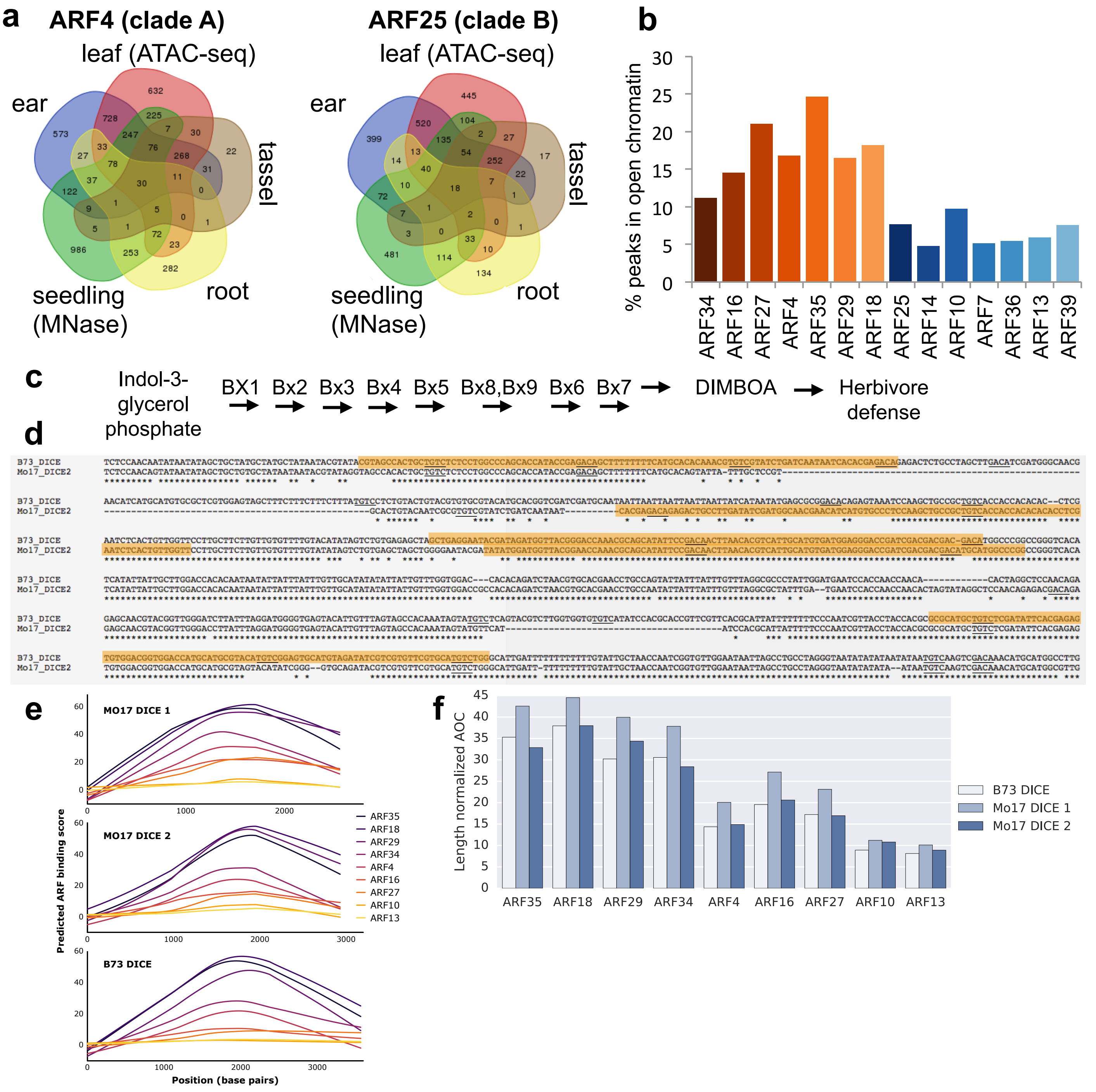

Supplementary Figure 12. ARFs bind to open chromatin regions of QTLs for domestication and herbivore resistance. a. Venn diagrams showing the number of ARF4 and ARF25 peaks that overlapped with regions of open chromatin from different tissues. **b**. Percentage of ARF peaks residing in regions of open chromatin from at least one of four different tissues (root, tassel, ear, and leaf). **c**. Schematic of DIMBOA biosynthesis pathway. **d.** Alignment of B73 DICE and Mo17 DICE 2 sequences surrounding the three ARF peaks. Orange highlighted regions show 100bp regions surrounding empirically determined peak summit. TGTC instances are underlined. Putative TGTC binding motifs in the Mo17 DICE2 region are partially conserved suggesting that ARF binding may play a role in the increased herbivore resistance of Mo17. **e.** Binding score predictions for individual ARFs in the DICE elements. **f**. Binding potential of individual ARFs to the B73 DICE, the Mo17 DICE1 and Mo17 DICE2 elements measured as the area under the curve (AOC) of binding scores within the ~4kb DICE elements. Values were normalized by length to account for the slightly different sizes of sequences between the different elements.

Supplementary Table 1. Table showing metrics for the 26 ARFs tested by DAP-seq. ARFs passing quality control threshold are in bold.

ZmARF	geneID	%RiP	# peaks	# target genes	# unique peaks	% of peaks that are unique	clade	sub-clade	% of ARF dataset that is subclade specific
**ARF34**	**GRMZM2G081158**	18	74686	9200	23786	32%	A	34/16/18	14%
**ARF16**	**GRMZM2G028980**	12	42142	6081	1746	4%	A	34/16/18	25%
**ARF27**	**GRMZM2G160005**	8	30731	5888	2378	8%	A	27/35	0.4%
**ARF4**	**GRMZM2G034840**	7	28732	4447	3786	13%	A	4/29	2%
**ARF29**	**GRMZM2G086949**	3	14201	2573	486	3%	A	4/29	4%
**ARF35**	**GRMZM2G317900**	2	9262	2139	342	4%	A	27/35	1%
**ARF18**	**GRMZM2G035405**	2	7544	1386	45	1%	A	34/16/18	5%
ARF9	GRMZM2G073750	0.6	2783	NA	NA	NA	A	NA	NA
ARF3	GRMZM2G078274	0.1	1122	NA	NA	NA	A	NA	NA
ARF22	GRMZM2G089640	0.1	303	NA	NA	NA	A	NA	NA
ARF30	GRMZM2G475882	0	89	NA	NA	NA	A	NA	NA
ARF1	GRMZM2G169820	0	21	NA	NA	NA	A	NA	NA
**ARF25**	**GRMZM2G116557**	17	38315	2789	5732	15%	B	10/13/25	18%
**ARF14**	**GRMZM2G137413**	16	28098	1640	2550	9%	B	14/36/39	4%
**ARF7**	**GRMZM2G475263**	11	29393	1561	8120	28%	B	7	29%
**ARF10**	**GRMZM2G338259**	10	33565	2711	2332	7%	B	10/13/25	19%
**ARF36**	**GRMZM2G702026**	9	21673	1288	772	4%	B	14/36/39	5%
**ARF13**	**GRMZM2G378580**	5	18360	913	164	1%	B	10/13/25	19%
**ARF39**	**GRMZM2G017187**	5	10196	747	46	0.5%	B	14/36/39	1%
ARF28	GRMZM2G006042	0.7	2699	NA	NA	NA	B	NA	NA
ARF12	GRMZM2G437460	0.5	1374	NA	NA	NA	B	NA	NA
ARF8	GRMZM2G352159	0	0	NA	NA	NA	B	NA	NA
ARF23	GRMZM2G441325	0	22	NA	NA	NA	C	NA	NA
ARF19	AC207656.3_FG002	0	22	NA	NA	NA	C	NA	NA
ARF5	GRMZM5G808366	0	0	NA	NA	NA	C	NA	NA
ARF21	GRMZM2G390641	0	0	NA	NA	NA	C	NA	NA

Supplementary Table 2. List of primers used in this study.

ARF	geneID	Forward primer	Reverse primer
ARF10	GRMZM2G338259	5’GAATTCGGCCGTCAAGGCCA**ATG CCGCCCGCAACAGCCAT**	5’AGTCGACGGCCCATGAGGCC**CTC GTTTGTGGCGGCGGCGCAA**
ARF12	GRMZM2G437460	5’GAATTCGGCCGTCAAGGCCA **ATGGCGGGCATCGACCTCAACACC**	5’AGTCGACGGCCCATGAGGCC**TTA CATATCTGTCGCCGAGACGTCAAC**
ARF13	GRMZM2G378580	5’GAATTCGGCCGTCAAGGCCA**ATG ACTTCTTCGGCGGCGG**	5’AGTCGACGGCCCATGAGGCC**GCA GTTGTCGGAGTAAGCGAC**
ARF39	GRMZM2G017187	5’GAATTCGGCCGTCAAGGCCA**ATG GACGCGCCCAACCCTGG**	5’AGTCGACGGCCCATGAGGCC**TTC AGACAGTGAGTTCGCAGC**

Supplementary Table 3. List of blacklisted sites with corresponding genomic coordinates (B73v3).

chromosome	start	end
1	234020	236940
1	8181420	8187000
1	18811180	18811780
1	23795660	23801280
1	24792560	24793320
1	25691580	25698100
1	188365200	188367240
1	203922040	203972020
1	234523860	234799180
1	247071880	247073040
1	277631740	277636580
1	281069480	281073800
1	288460640	288522680
2	7876796	7879820
2	65623040	65634920
2	78451820	78533960
2	172722480	172728660
2	172753000	172758620
2	174735560	174738860
2	229751620	229788080
3	29367300	29370920
3	50972180	50973720
3	50972507	50973820
3	74665400	74669220
3	81184180	81201060
3	165555760	165557740
3	215294200	215294520
4	74593940	74598580
4	85001080	85001840
4	111648720	111735480
4	123491547	123515060
4	156783640	156803120
4	156978620	157019540
4	172406460	172456480
4	208416120	208416460
5	21523600	21527900
5	147741620	147742880
5	189292040	189293760
5	209989400	210066300
6	51095820	51108200
6	51591260	51601900
6	68902720	68903380
6	159151460	159151960
6	160952600	160983300
7	37451180	37456540
7	44740920	44744580
7	63569200	63570020
7	129594460	129670380
8	17386620	17387060
8	135509580	135512680
8	145996600	145997200
8	155678640	155702560
8	175341900	175345520
9	1	123900
9	6936360	6968600
9	28874200	28875280
9	58974640	59006800
9	68987360	69066640
9	68987680	69065480
9	69842480	69848160
9	73732140	74000800
9	88177100	88204100
9	132009360	132010480
10	121020780	121028520

Supplementary References

1. Boer, D. R. *et al.* Structural basis for DNA binding specificity by the auxin-dependent ARF transcription factors. *Cell* **156,** 577–589 (2014).

2. Guilfoyle, T. J. The PB1 Domain in Auxin Response Factor and Aux/IAA Proteins: A Versatile Protein Interaction Module in the Auxin Response. *Plant Cell Online* **27,** 33–43 (2015).

3. Walley, J. W. *et al.* Integration of omic networks in a developmental atlas of maize. *Science* **353,** 814–8 (2016).
